# Supplementary material for: COZOID: contact zone identifier for visual analysis of protein-protein interactions
Source: BMC Bioinformatics. 2018 Apr 6;19:125. doi: 10.1186/s12859-018-2113-6 (PMC5889581; doi:10.1186/s12859-018-2113-6)
Supplement: Supplementary file 2 — Software build. Executable binary file of the software tool. (ZIP 118,784 kb) [file 12859_2018_2113_MOESM2_ESM.zip › cozoid/java/docs/beansbinding-1.2.1-doc.zip!/org/jdesktop/beansbinding/package-frame.html]

org.jdesktop.beansbinding (Beans Binding)


org.jdesktop.beansbinding

Interfaces 
  
*BindingListener*
  
*PropertyStateListener*

Classes 
  
AbstractBindingListener
  
AutoBinding
  
BeanProperty
  
Binding
  
Binding.SyncFailure
  
Binding.ValueResult
  
BindingGroup
  
Bindings
  
Converter
  
ELProperty
  
ObjectProperty
  
Property
  
PropertyHelper
  
PropertyStateEvent
  
Validator

Enums 
  
AutoBinding.UpdateStrategy
  
Binding.SyncFailureType

Exceptions 
  
PropertyResolutionException
